# Liver Macrophage Depletion Ameliorates The Effect of Mesenchymal Stem Cell Transplantation in a Murine Model of Injured Liver

**DOI:** 10.1038/s41598-018-37184-4

**Published:** 2019-01-10

**Authors:** Lobna y. Ghanem, Iman M. Mansour, Nelly Abulata, Maha M. Akl, Zeinab A. Demerdash, Hanan G. El Baz, Soheir S. Mahmoud, Salwa H. Mohamed, Faten S. Mahmoud, Ayat S. M. Hassan

**Affiliations:** 1 Departments of Electron Microscopy, Theodor Bilharz Research Institute, Warrak El-Hadar, Imbaba, P.O. Box 30, Giza, 12411 Egypt; 2 Department of Immunology, Theodor Bilharz Research Institute, Warrak El-Hadar, Imbaba, P.O. Box 30, Giza, 12411 Egypt; 3 Department of Pathology, Theodor Bilharz Research Institute, Warrak El-Hadar, Imbaba, P.O. Box 30, Giza, 12411 Egypt; 4 Department of parasitology, Theodor Bilharz Research Institute, Warrak El-Hadar, Imbaba, P.O. Box 30, Giza, 12411 Egypt; 5 Department of Clinical & Chemical pathology, Kasr Al-Ainy hospital, Faculty of medicine, Cairo University, Cairo, 11562 Egypt

## Abstract

Mesenchymal stem cells (MSCs) therapy show different levels of effectiveness in the context of different types of liver damage, suggesting that the microenvironment of the injured liver is a key determinant for effective stem cell therapy. The objective was to assess the modulatory effect of hepatic stem cell niche components on the transplanted MSCs during liver injury induced by carbon tetrachloride (CCl_4_). Superparamagnetic iron oxide (SPIO)-labeled human MSCs were injected intravenously into mice treated with CCl_4_ and subjected to hepatic macrophage-depletion. Liver tissues were collected at different intervals post transplantation for subsequent histopathological, morphometric, immunohistochemical, gene expression and ultrastructural studies. The homing of the transplanted MSCs was evidenced by tracing them within the niche by iron staining and immunohistochemical studies. MSCs differentiated into hepatocyte-like cells and intimal smooth muscle cells as evidenced by their expression of human albumin and α-smooth muscle actin with a concomitant increase in the level of mouse hepatocyte growth factor. A post transplantation reduction in the liver fibro-inflammatory reaction was found and was promoted by liver macrophages depletion. Thus, it could be concluded from the present study that prior manipulation of the microenvironment is required to improve the outcome of the transplanted cells.

## Introduction

A stem cell niche is a limited compartment in a tissue keeping up and controlling stem cell behaviour, supporting self-renewal and keeping the harmony between the state of quiescence, proliferation and differentiation in response to tissue damage^1^.

The hepatic stem cells microenvironment includes the extracellular matrix (ECM), epithelial and non- epithelial resident liver cells, and recruited inflammatory cells as well as a variety of cytokines. Stem cell interacts with various inputs from the microenvironment such as soluble factors, ECM and intercellular contacts acting together to influence stem cell fate^2^.

Non-parenchymal cells (NPCs) in the liver include stellate cells/myofibroblasts, which are the principle source of collagen; macrophages, which are involved in tissue remodelling and fibrosis resolution after tissue damage^3^; endothelial cells, which are capable of new vessels formation and other recruited inflammatory cells. NPCs produce cytokines and growth factors, like transforming growth factor β (TGF β), that have a major effect on hepatic progenitor cells (HPCs) and hepatocyte proliferation^4^.

Macrophages act at different sites of inflammation, participating in the process of inflammation and repair, demonstrating different activities such as regulation of inflammatory cells, tissue debridement, recruiting and activating myofibroblasts, and regulating spontaneous recovery of fibrosis in wound healing. These functions of macrophage are mediated through secretion of cytokines, like TGF-β and tumour necrosis factor- α TNF-α^3^.

Recent studies demonstrate a role of macrophage in modulating the hematopoietic stem cell niche, in which reductions in bone marrow (BM) mononuclear phagocytes led to reduced BM stromal derived factor-1(SDF-1) levels, the selective down-regulation of hemopoietic stem cell retention genes in niche cells, and mobilization of hemopoietic stem cells to the blood stream^5^. Furthermore, evidences from other studies suggesting a role of the inflammatory cytokine TGF-β in regulating stem cell behaviour^6,7^.

Increased expression of hepatocyte growth factor (HGF) has been demonstrated in carbon tetrachloride (CCl_4_)-induced liver injury and in rodent hepatic oval cell regeneration models, suggesting its involvement in stem cell proliferation, migration, and differentiation^8^.

On the basis of the previous studies, understanding how the niche influences stem cells behaviour is therefore important for scientific and clinical issues for promoting liver regeneration in chronic liver injury. As Macrophages could be considered as a key regulator of the liver stem cell niche, so inducing macrophage depletion from the liver will be important to assess their role in the context of stem cell transplantation in animal models with liver fibrosis.

The aim of the present study was to determine the behaviour of transplanted MSCs within the injured liver in response to its interaction with niche components, particularly macrophage. To achieve our aim, human MSCs were injected intravenously into mice treated with CCl_4_ and subjected to hepatic macrophage-depletion. Livers were collected at different intervals post transplantation for subsequent histopathological, morphometric, immunohistochemical, gene expression and ultrastructural studies.

## Methods

### Animals

The present study was conducted on 90 Swiss albino male mice with liver injury induced by carbon tetrachloride (CCl_4_). The control group comprised 30 age-matched and sex-matched healthy mice. All mice were aged 4 weeks and weighed an average of 20 ± 5 g. The animals were obtained from the Schistosoma Biological Material Supply Center of the Theodor Bilharz Research Institute (TBRI), Giza, Egypt. They were housed under conventional conditions and fed a standard diet with free accessibility to water. All animal work was conducted in accordance with the guidelines outlined in the Guide for the Care and Use of Laboratory Animals and was approved by the Research Ethics Committee of the Theodor Bilharz Research Institute, Giza, Egypt.

### Experimental design

The mice were randomly allocated into eight groups containing 15 mice each as follows:

**Group I:** Healthy control group, **Group II:** Healthy group transplanted with Superparamagnetic iron oxide (SPIO)-labeled MSCs, **Group III:** A group with mild liver fibrosis induced by CCl_4_, **Group IV:** A group with mild liver fibrosis induced by CCl_4_ with hepatic macrophage depletion and transplanted with SPIO-labeled MSCs, **Group V:** A group with mild liver fibrosis induced by CCl_4_ that was nondepleted and transplanted with SPIO-labeled MSCs, **Group VI:** A group with marked liver fibrosis (Cirrhosis) induced by CCl_4,_ **Group VII:** A group with cirrhosis induced by CCl_4_ with hepatic macrophage depletion and transplanted with SPIO-labeled MSCs, and **Group VIII:** A group with cirrhosis induced by CCl_4_ that was nondepleted and transplanted with SPIO-labeled MSCs.

### Induction of hepatic injury

#### Mild liver fibrosis

Intra-peritoneal (IP) injection of 10 µl/g body weight (BW) of 10% CCl_4_ (in corn oil) three times/week for 5 weeks.

#### Cirrhosis

IP injection of 10 µl/g BW of 20% CCl_4_ (in corn oil) three times/week for 12 weeks (modified from Duffield JS *et al*.^3^).

Grading of liver damage was done using the fibrosis-scoring system of Ishak *et al*.^9^.

### Depletion of liver macrophages

After induction of liver fibrosis, mice were administered an intravenous (IV) injection of 0.1 ml/mouse (30 g BW) of either clodronate-containing liposomes (for the fibrosis and cirrhosis groups) or empty liposomes (for the control group) (Encapsula Nanosciences, LLC) approximately 48 hr before MSC transplantation. After 2 days, the liver macrophages were depleted as previously described^10^. Depletion was confirmed by immunohistochemical analysis using the Anti f4/80 antibody for mouse macrophages.

### Isolation and culture of MSCs from umbilical cord blood (UCB)

The MSCs used in the present study were originally isolated and expanded from a donated human umbilical cord with the donor’s consent after a full-term cesarean delivery. The cells obtained were cultured in complete culture medium comprising low-glucose Dulbecco’s modified Eagle medium (DMEM; Sigma-Aldrich Co.,USA) supplemented with 1% L-glutamine, 30% fetal calf serum (FCS; Sigma-Aldrich Co., USA) and 1% penicillin-streptomycin (Invitrogen, USA). The cells were collected between the third and fourth passages for further transfusion into mice.

### MSC labeling by SPIO

Isolated MSCs from the third passage were labeled with a combination of superparamagnetic iron oxide (SPIO) (Nanotech, Egypt) and poly-L-lysine (PLL, Sigma), as previously described^11^. Briefly, cultured MSCs were incubated with the labeling medium (DMEM containing 50 μg/ml iron and 0.75 μg/ml PLL) for 48 h at 37 °C with 5% CO_2_ at a density of 3 × 10^5^ cells/ml. Control MSCs were cultured in DMEM without iron or PLL. After cell recovery, the cells were either harvested by trypsin digestion or examined *in situ* for viability and iron staining. As outlined below, Perls’ Prussian blue staining (ferric hexacyanoferrate and hydrochloric acid, Sigma) and transmission electron microscopy were both used to assess iron staining.

### Transmission electron microscopic examination of SPIO-labeled MSCs

Immediately following the labeling procedure, an aliquot of the labeled cells was fixed for 30 min in 2.5% glutaraldehyde in PBS buffer at room temperature. The cells were then washed three times in PBS and postfixed for 30 min in 2% osmium tetroxide in PBS buffer. After dehydration in 4 changes of graded ethanol (50%, 70%, 90%, and 100%) with a 5 min gap, they were embedded in Epon 812 substitute (TAAB Laboratories Equipment Ltd., Aldermaston, Berkshire, UK) and polymerized at 60 °C for 48 h. Semi-thin sections were cut, stained with methylene blue-azure II, and examined by light microscopy to choose the region of interest for ultrathin sectioning. The ultrathin sections were then prepared using an Ultracut R ultramicrotome (Leica, Vienna, Austria), double stained with uranyl acetate and lead citrate and examined with a Philips EM 208 S electron microscope (Philips Optics, Eindhoven, The Netherlands).

### Transplantation of SPIO-labeled MSCs

After 5 weeks and 12 weeks of CCl4 injection, 1 × 10^6^ SPIO-labeled MSCs per mouse were injected intravenously in the tail vein (1 × 10^6^ cells/100 µl).

### Sacrifice of mice and collection of liver samples

Mice were sacrificed at different time intervals post transplantation. Liver samples obtained at 24 h were used for the assessment of homing, whereas those obtained at 12 weeks were used to assess the engraftment of transplanted MSCs. The liver samples were subsequently processed using histopathologic, ultrastructural, immunohistochemical and quantitative real-time polymerase chain reaction (RT-PCR) techniques.

### Histopathology

Excised livers were immediately fixed in a 10% formalin solution and embedded in paraffin. They were then processed and stained with hematoxylin and eosin (H&E) stain to examine histopathologic changes. Masson’s trichrome stain was used to qualitatively assess the grade of fibrosis.

### Morphometric Studies

Hepatic tissue sections (5 μm in thickness) were prepared and stained with Sirius red (SR) to quantify collagen content using a Zeiss Scope A1 microscope equipped with a CCD camera (Axiocam MRC5). Image analysis was performed using the Axiovision Rel.4.8 computer software.

### Immunohistochemistry (IHC)

Immunohistochemistry was performed using an avidin–biotin complex immunoperoxidase technique. Human-specific antibodies with no cross-reactivity to mouse antigens were used to label the following markers: α smooth muscle actin (α-SMA, Cat. No. NB600-536, Novusbio, USA); albumin (Cat. No. NBP2-21669, Novusbio, USA); and Von Willebrand factor (Cat. No. NB600-586, Novusbio, USA). The anti-OxPhos complex IV subunit I monoclonal antibody (Cat. No. 459600, Invitrogen, USA) and the rabbit monoclonal Anti-f4/80 mouse antibody (Cat. No. AB111101, Abcam) were also used. We used a preformed streptavidin–biotin–peroxidase complex and peroxidase-DAB (3,3′-diaminobenzidine) (Dako, Denmark) according to the manufacturer’s instructions. Sections were counterstained with Mayer’s hematoxylin and mounted using DPX medium. Positive and negative control slides for each marker were included in each run. As a negative control, a liver tissue section was processed as described but with the primary antibody omitted.

### Locating the stem-cell niche in the liver tissue by IHC

A technique for lineage tracing in liver tissue was used following Fellous *et al*.^12^. Briefly, IHC staining using the anti-OxPhos complex IV subunit I monoclonal antibody (Invitrogen) (dilution 1:50) for detecting patches of cytochrome *c* oxidase-negative hepatocytes in liver invariably connected to the portal areas suggested an origin from a long-lived cell, presumably a stem cell.

### Homing assay for transplanted SPIO-labeled MSCs in the liver

#### Prussian blue staining of the liver tissue

At 24 h after MSC transplantation, following location of the niche by IHC, serial sections from the same paraffin block were used for Prussian blue staining as previously described^13^. The homing of the transplanted MSCs into the recipient liver was estimated semi-quantitatively by Prussian blue staining and counting the number of periportal regions (within the same cytochrome *c* oxidase–negative patches) by considering the bluish coloration in 5 consecutive positive high-power fields.

### Gene expression analysis

Gene expression analysis was performed using quantitative real-time polymerase chain reaction (qPCR). The total RNA was extracted and purified from liver tissue using the RNeasy Protect Mini Kit (Cat. No. 74124, Qiagen, USA) according to the manufacturer’s instructions. Complementary DNA was synthesized by reverse transcription (RT) of the RNA using the QuantiTect RT Kit (Qiagen, USA, Cat. No. 205310). Specific bioinformatically validated primers were provided by Qiagen for the quantification of mouse HGF (Cat. No. QT00158046), mouse TGFB1 (Cat. No. QT00145250) and mouse SDF1 (CXCL12) (Cat. No. QT00161112). The expression of mRNA was assessed by qPCR using the QuantiTect SYBR Green PCR kit (Cat. No. 204143 Qiagen, USA) and an ABI PRISM 7500 sequence detector (Applied Biosystems, CA, USA). The quantification was performed using the comparative Cq method as previously described^14^. The level of expression was normalized to the reference gene, glyceraldehyde 3-phosphate dehydrogenase (GAPDH) (Cat. No. QT01658692).

### Statistical Analysis

The data were analyzed using version 22 of the SPSS statistic software (IBM Corp., Armonk, NY, USA). The data were expressed as the median and percentiles for quantitative non-parametric measures. The following tests were performed: the Wilcoxon Rank Sum test for comparisons between two independent groups for non-parametric data; the ranked Spearman correlation test to study the possible association between two variables among each group for non-parametric data; and Kruskal–Wallis test for comparisons between more than two groups for non-parametric data. A probability of error (P value) less than 0.05 was considered significant.

## Results

### SPIO-labeling of MSCs

**Figure 1:**
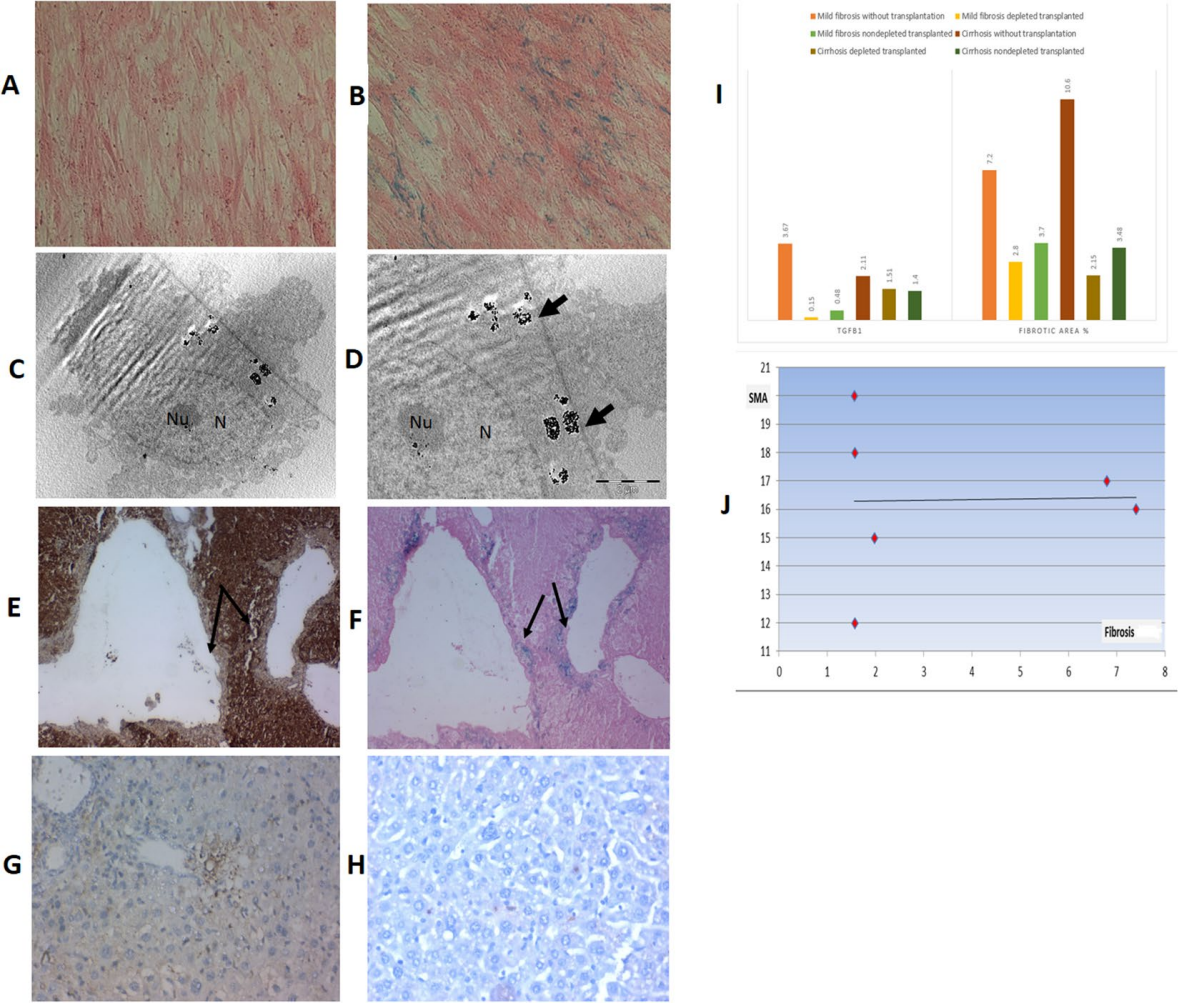
Cultured and Labelled mesenchymal stem cells morphology by light microscope without superparamagnetic iron oxide (SPIO) labeling (x40) (**A**) and with SPIO labeling after staining with Prussian blue (x40) (**B**). Transmission electron microscopic examination (**C**) x5600 & (**D**) x11000, showing labeled cells with iron particles appear as black aggregates among numerous round endosomes within the cytoplasm (arrows). The nucleus (N) is convoluted, with prominent euchromatin and peripheral heterochromatin, a conspicuous nucleolus (Nu) is also seen. Homing of the SPIO-labelled transplanted MSCs into their niche evident by immunohistochemical (IHC) staining (**E**) & Prussian blue staining (**F**) of the recipient liver where IHC using the anti-OxPhos complex IV subunit I monoclonal antibody showing areas that were negative for cytochrome C oxidase located either in close proximity to, or in direct contact with, the portal tract region (arrows; X40 magnification). Prussian blue staining showing bluish iron particles distributed intracellularly in the same region as the cytochrome C oxidase-deficient patches (arrows; X40 magnification). Successful macrophage depletion from the liver shown by IHC, the normal distribution of Kupffer cells is evident in nondepleted liver (**G**) with significant reduction after using clodronate liposome with very few positive anti f4/80 ab cells in the hepatic parynchyma x40 (**H**). (**I**) Comparison between all fibrosis and all cirrhotic groups regarding fibrosis level and TGFb1 level (**J**) The correlation between αSMA expression and the fibrosis grade after MSC transplantation. As shown, no correlation could be detected.

MSCs isolated and propagated in cultured media formed adherent cell layers of spindle-shaped cells with a confluency of 70–90%. Viability of these cells was 90–95%. The cells were assessed for efficient labeling by SPIO using Perls’ Prussian blue staining and ultrastructural studies where the presence of bluish iron particles was visualized by an inverted microscope. Most of the MSCs treated with SPIO displayed cytoplasmic staining when compared to the control (unlabelled) MSCs (Fig. 1A,B).

The electron microscopic examination of the labeled MSCs confirmed the internalization of SPIO nanoparticles by the cells rather than simply their bonding to the cell surface. The SPIO nanoparticles appeared as black aggregates in numerous round endosomes in the cytoplasm and perinuclear area, while cell membrane-bound nanoparticle clusters were not detected (Fig. 1C,D).

### Locating the stem cell niche and tracing the homing of the transplanted MSCs within the liver

IHC staining using the anti-OxPhos complex IV subunit I monoclonal antibody detected patches of cytochrome C oxidase-negative areas located in close proximity to or in direct contact with the portal tract region. The patches varied in size but had clearly defined boundaries, seemingly always extending to the peri-venous regions (Fig. 1E). Tracing the homing of human cells into recipient mouse livers was confirmed by Prussian blue staining of the liver tissue (Fig. 1F) obtained at 24 h post-transplantation. The presence of bluish iron particles was visualized intracellularly in the same region of the cytochrome C oxidase-deficient patches in liver tissue, confirming that the transplanted MSCs homed to their niche in the region of the portal tract and perivascular regions.

### Effect of macrophage depletion on the outcome of transplanted MSCs

Successful hepatic macrophage depletion was confirmed by immunohistochemical analysis using the Anti f4/80 antibody for mouse macrophages Fig. 1(G,H).

#### Reduction of liver fibro-inflammatory reaction

**Figure 2:**
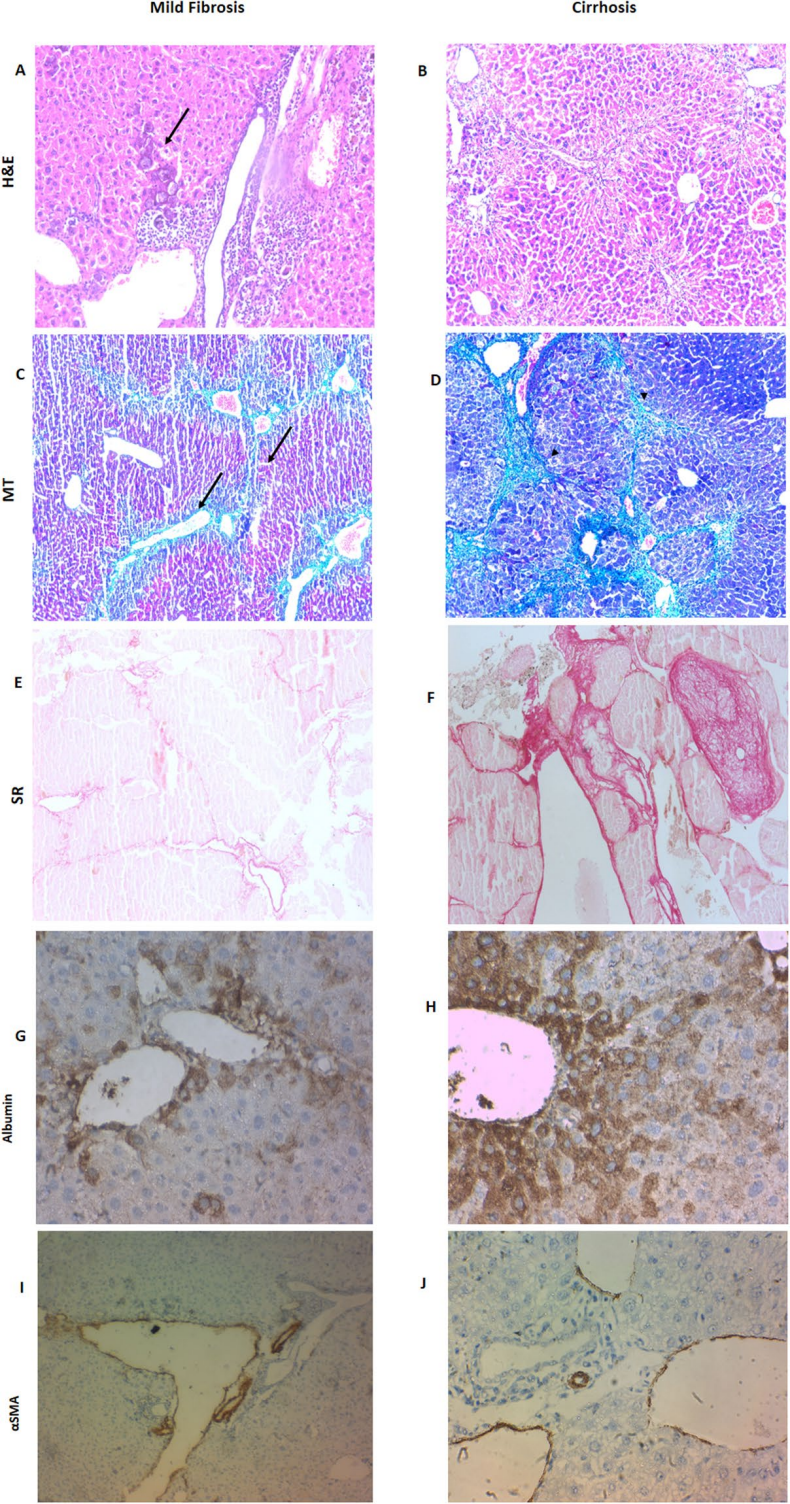
Photomicrographs for liver sections of mild liver fibrosis and cirrhosis groups stained with haematoxylin and eosin (H&E, X10) (**A**,**B**), Masson trichrome (MT, X10) (**C**,**D**) and Sirius red staining (SR,X10) (E&F), as well as immunohistochemical staining (immunostain, DAB, X40) of human albumin (**G**,**H**) and human α smooth muscle actin (αSMA) (**I**,**J**). In the H&E, MT and SR-stained sections, the fibrotic areas was significantly less with perivascular distribution and mild hepatic degeneration (arrow) and mononuclear cells infiltration in the mild fibrosis groups, where a marked fibrosis with cirrhotic nodules (arrows head), hepatocyte degeneration and loss of the hepatic architecture was observed in the cirrhotic group. As for the immunostained sections, Human albumin is shown in small clusters of polygonal (hepatocyte-like) cells with cytoplasmic expression. Note the higher expression in cirrhotic (**H**) *vs*. mild fibrosis (**G**), where for αSMA (**I**,**J**),human cells that stained positive for this marker are distributed perivascularly with thickened vessel walls (neointima formation).

To determine whether MSC transplantation reduces liver fibrosis in macrophage-depleted mice, we administered MSCs intravenously to cirrhotic or fibrotic mice and assessed liver fibrosis in untreated mice. Liver fibrosis assessed by H&E, SR and Masson’s trichrome staining (Fig. 2).

Histopathological examination of the recipient mouse liver during different stages of liver damage (both mild fibrosis and cirrhosis) revealed a statistically significant reduction in the percentage of the assessed fibrotic area after MSC transplantation and macrophage depletion compared to the nontransplanted group, as shown by SR staining, and that the reduction in the percentage of the assessed fibrotic area was favored by the depletion of liver macrophages (Fig. 1I). Moreover, there was a reduction in fibrosis after transplantation without macrophage depletion but not reaching a statistically significant level, as shown in Table 1. Taken together, these results suggest that MSCs home to the liver and decrease fibrosis in a mouse model of cirrhosis However, a more significant fibrosis reduction occurred in the transplanted group in which macrophages were depleted in comparison to the corresponding group without macrophage depletion.

**Table 1:** Effect of hepatic macrophage depletion on the therapeutic potential of the transplanted human mesenchymal stem cells among all cases of mild fibrosis and cirrhosis groups in comparison to the corresponding groups without transplantation regarding the differentiation into hepatic cells expressing human albumin & α smooth muscle actin (αSMA), and the reduction of fibrosis with concomitant changes in the level of expression of mouse transforming growth factor b1 (TGFb1), mouse hepatocyte growth factor (HGF) and mouse stromal derived factor 1(SDF1).

Parameter	Mild fibrosis without transplantation	Mild fibrosis depleted transplanted	Mild fibrosis nondepleted transplanted	P value	Cirrhosis without transplantation	Cirrhosis depleted transplanted	Cirrhosis nondepleted transplanted	P value
TGFb1	3.67 (a)	0.15 (b)	0.48 (c)	<0.001	2.11 (a)	1.51 (a)	1.40 (a)	0.400
HGF	0.91 (a)	0.56 (a)	0.56 (a)	0.600	0.90 (a)	1.33 (b)	1.49 (c)	0.020*
SDF1	0.53 (a)	0.56 (a)	0.98 (a)	0.500	0.90 (a)	1.90 (b)	1.31 (c)	0.001*
Fibrotic area %	7.20 (a)	2.80 (b)	3.70 (a)		10.60 (a)	2.15 (b)	3.48 (a)	
αSMA	NA	35.50 (a)	26.50 (b)	0.016*	NA	38 (a)	16.50 (b)	0.005*
Albumin	NA	10.60 (a)	5.70 (b)	0.004*	NA	17 (a)	7.95 (b)	0.006*

Values are given as the median, unless otherwise indicated.

*A P value < 0.05 was considered statistically significant.

Groups sharing same letter were not significantly different.

Depleted means groups subjected to hepatic macrophage depletion, nondepleted means groups not subjected to hepatic macrophage depletion, NA: Not applicable.

#### Engraftment and differentiation of MSCs by IHC studies

MSCs differentiated into hepatocyte-like cells as evidenced by their expression of human albumin. The expression of human albumin in small clusters of polygonal cells (as newly formed hepatocyte-like cells) was cytoplasmic, distributed in small clusters near the portal areas, perivascular and scattered intralobularly in the hepatic parenchyma, as shown in Fig. 2(G,H). Human αSMA expression was chosen primarily to be assessed as a marker for activated hepatic stellate cells that play a central role in the pathogenesis of hepatic fibrosis. However, we found that MSCs differentiated into intimal smooth muscle cells expressing human αSMA which were distributed mainly perivascularly within the thickened vessels wall, as shown in Fig. 2(I,J), and there was an absence of any correlation between αSMA expression and fibrosis (Fig. 1J).

The lack of endothelial differentiation of MSCs was reported in our study as evidenced by absence of expression of human VWF in both fibrotic and cirrhotic livers.

The expression of human αSMA and albumin after MSC transplantation was statistically significantly higher in macrophage-depleted animal groups compared to the nondepleted groups, as shown in Table 1. Generally, this expression was significantly higher and enhanced in the cirrhosis stage compared to the mid-fibrosis stage, as shown in Table 2.

**Table 2:** Between-group comparisons of human albumin and α smooth muscle actin (αSMA) expression.

Parameter	Cirrhosis depleted transplanted	Mild fibrosis depleted transplanted	P value	Cirrhosis nondepleted transplanted	Mild fibrosis nondepleted transplanted	P value
αSMA	38.00	35.50	0.004*	16.50	26.50	0.070
Albumin	17.00	10.60	0.004*	7.95	5.70	0.030*

Values are given as the median, unless otherwise indicated.

*A P value < 0.05 was considered statistically significant.

Depleted means groups subjected to hepatic macrophage depletion, nondepleted means groups not subjected to hepatic macrophage depletion.

### Gene expression analysis

The results showed that the relative mRNA expression level of mouse TGFb1 was increased after CCl4-induced liver fibrosis compared to the baseline value in the control group and was not changed significantly in cirrhosis compared to the control (Table 3). However, these values were significantly reduced after MSC transplantation in the mild fibrosis group (both depleted and non-depleted) compared with that in the mild fibrosis group without transplantation. The significant decrease in the TGFb1 level was greater in the macrophage-depleted group than in the non-depleted group, as shown in Table 1. However, there was no significant difference detected among all groups of cirrhosis before and after transplantation compared to each other (cirrhosis, cirrhosis depleted transplanted and cirrhosis non-depleted transplanted) (Table 1).

**Table 3:** Between-group comparisons of mouse transforming growth factor b1 (TGFb1), mouse hepatocyte growth factor (HGF) and mouse stromal derived factor 1(SDF1) and Fibrotic area %.

Parameter	Control normal healthy mice	Mild fibrosis	P value	Control normal healthy mice	Cirrhosis	P value
TGFb1	1.07	3.67	0.004*	1.07	2.11	1.000
HGF	0.70	0.91	0.170	0.70	0.90	0.08
SDF1	0.44	0.53	0.420	0.44	0.90	0.08
Fibrotic area %	0.23	7.20	0.001*	0.23	10.60	0.001*

Values are given as the median, unless otherwise indicated.

*A P value < 0.05 was considered statistically significant.

The relative mRNA expression levels of mouse HGF and SDF1 did not change significantly among all groups of mild fibrosis before and after transplantation compared to each other (Table 1). However, in the cirrhosis groups, the relative expression of SDF1 and HGF increased after MSC transplantation in both the depleted and non-depleted groups compared to the corresponding nontransplanted groups (Table 1).

## Discussion

On the basis of the recently recognized potential of MSCs to contribute to tissue repair after injury in different diseases including heart and liver diseases^15–17^, MSC transplantation has recently gained widespread enthusiasm to use them to treat acute and chronic liver diseases. However, controversy has arisen as various situations of liver damage could affect the potential of MSCs to show a full beneficial effect^18–20^. This suggested that the microenvironment of the injured liver is a key consideration for effective stem cell therapy. In the current study, experimental animal models were designed to identify the behaviour of transplanted stem cells in response to liver damage and their relationship to hepatic stem cell niche. Super paramagnetic iron oxide (SPIO) - labelled MSCs were injected into mice in which liver fibrosis by carbon tetrachloride was induced. To identify the role of macrophages as one cellular component of the niche, selective hepatic macrophage-depleted animal model subgroup was used using clodronate liposome. Transplanted labelled stem cells are then traced for their homing into their niche and were studied for their differentiation into hepatocytes, neovasculature or myofibroblasts and these activities were correlated with various levels of soluble factors released in the stem cell niche compartment at early and late stages of liver fibrosis.

Our findings demonstrated efficient homing of transplanted MSCs into the recipient liver of injured mice, with a higher effeciency in cirrhotic livers than in the mildly fibrosed livers compared to normal mice transplanted with the same MSCs, which did not show homing of these cells.

Our findings are in agreement with those of both Seo *et al*.^21^ and Sato *et al*.^22^. These researchers reported that labelled human MSCs were retrieved from the liver of CCl_4_-injured mice after intravenous and intrahepatic injection and they had differentiated into hepatocyte-like cells.

The absence of human cells in the liver of control mice following IV transplantation of MSCs observed in our study is in agreement with the findings of Wagers *et al*.^23^. These investigators found that transplanted hematopoietic stem cells differentiate into hepatocytes at a very low level in mice without liver injury, suggesting that both liver injury and the surrounding environment could be essential for transplanted cells to recruit to the liver and to differentiate into hepatocytes. These factors were explained later in studies suggesting that stromal cell-derived factor 1 (SDF1) and its receptor CXCR4 might be important for stem-cell recruitment to the liver^24^.

Our results revealed that SDF1 level was statistically significantly higher in all cirrhosis groups post transplantation (week 12) with more increase in depleted transplanted group compared to the non-transplanted counterparts, however no statistically significant difference was found in the mild fibrosis groups. Similarly, recent study demonstrated that the SDF-1/CXCR4 axis play an important role in the chemotaxis, homing, and differentiation of MSCs to the injured liver^25^.

Several possibilities could explain the lack of increase of SDF1 levels in all of the mild fibrosis groups and its marked increase in the cirrhotic depleted transplanted group. These explanations are in line with the view of Di Campli *et al*., who attributed the lack of migration of circulating stem cells from the bone marrow to the liver in patients with a variety of chronic hepatic injuries to the fact that there could be a balance between injury and endogenous repair mechanisms, excluding the need for extra hepatic progenitors, in a mildly damaged liver^26^. While the extrahepatic BM-derived stem cells could only come into action when the body’s repair mechanisms are overwhelmed, as occurs in severe cirrhosis^27^_._ This could be supported by our finding of a concomitant increase in the number of engrafted and differentiated cells in the cirrhotic transplanted group in relation to the mild fibrosis group.

Several studies have shown both antifibrotic and anti-inflammatory effects of MSCs derived from several sources. They related this protective effect to the ability of MSCs to decrease the level of inflammatory cytokines, such as IL-1β, IL-6, TNF-α and TGF-β^28,29^. Additionally, other studies suggested that MSCs could exert their anti-fibrotic effects through the secretion of matrix metalloproteinases^18,30^. However, some studies failed to show the beneficial effect of MSCs in reducing the fibro-inflammatory reaction, as they conversely found that MSCs might contribute to fibrosis production. They suggested that this might be related to either the various subpopulations of MSCs with opposing effects or the type of liver injury and extent of fibrosis^19,20,31^.

In our study, the histopathological picture of early mild fibrosis animal group revealed a mild fibro-inflammatory picture evidenced by MNCs infiltration and mild perivascular fibrosis in the portal area and around central veins, and more fibrosis with loss of hepatic lobular architecture was present in the cirrhotic group as represented by Masson trichrome stain and Sirus red stain analysis. Twelve weeks later, we correlated the changes that took place after MSCs transplantation in comparison to the corresponding group without transplantation. Our findings revealed that there was a statistical significant reduction in liver fibro-inflammatory reaction after MSC transplantation (2.80 for mild fibrosis depleted transplanted *vs*. 7.20 for mild fibrosis without transplantation, p = 0.005) and (2.15 for cirrhotic depleted transplanted *vs*. 10.6 for cirrhotic non transplanted, p < 0.001). In spite of the observed reduction in the liver fibro inflammatory reaction after MSC transplantation in both mild fibrosis and cirrhosis groups without macrophage depletion, yet this reduction did not reach statistical significance (P value 0.7 and 0.4 respectively) Table (1). Thus our findings demonstrated that MSC behavior could be affected by the surrounding microenvironment as evidenced by the potentiation of the anti-fibrotic and anti-inflammatory effects of transplanted MSCs when the microenvironment was depleted of the hostile macrophage element. Previous studies have reported the inflammatory and fibrogenic role of macrophages in liver diseases^32^. This pro-fibrotic effect of macrophages might contribute to the negative effects of transplanted MSCs in the nondepleted transplanted group demonstrated in our study. Hepatic macrophages might induce liver fibrosis by promoting stellate-cell activation in the presence of continued injury through TGFb1 paracrine stimulation^3^. They were also proved to interfere with fibrosis degradation via increasing expression of tissue inhibitors of metalloproteinase -1(TIMP-1) from the stellate cell^32^. This is supported in our study by the substantial reduction in the TGFb1 level in the mild fibrosis depleted transplanted vs. mild fibrosis nondepleted transplanted group (p value < 0.001).

In our study, transdifferentiation of transplanted MSCs was evidenced by presence of human albumin-positive hepatocyte-like cells. Furthermore, the expression of human albumin cells was higher in the cirrhotic transplanted group than in the mild fibrosis transplanted group and more in the macrophage-depleted groups than in the nondepleted groups.

Previous studies have reported that in severe fibrotic or cirrhotic livers, the number of hepatocytes is substantially reduced, and the transdifferentiation of donor MSCs to parenchymal hepatocytes has been repeatedly demonstrated *in vivo* when transplanted during acute and chronic liver injury^22,33^. These studies have proved that in a mildly damaged liver, there could be a balance between injury and endogenous repair mechanisms, excluding the need for extra hepatic progenitors, which could only come into action when the body’s repair mechanisms are overwhelmed, as in severe cirrhosis^27^.

Our results revealed that in untransplanted animals, HGF expression was not significantly different from the baseline values of the controls in both fibrosis and cirrhosis groups. However, the level of HGF increased significantly after MSC transplantation in cirrhotic animals, both in the macrophage-depleted and nondepleted groups. Similarly, Tsai and Prosser *et al*. have demonstrated that MSC transplantation increased levels of HGF, which in turn induced MSC trans differentiation into parenchymal hepatocytes^28,34^.

In an attempt to investigate the angiogenic role of MSCs as part of reparative potential in liver disease, the transdifferentiation of transplanted MSCs in the injured liver to endothelial cells was examined by their hVWF expression. However, our results revealed no expression of hVWF in both the fibrotic and cirrhotic conditions following MSC transplantation. This could suggest that MSCs do not participate in angiogenic remodeling during liver regeneration. The lack of endothelial differentiation of MSCs was also reported in another study^35^.

In addition to the beneficial reparative effect of MSCs in liver disease, unwanted effects such as myofibroblastic transdifferentiation was reported^4^. Therefore, in the present study, the human αSMA expression was primarily assessed as a marker for activated hepatic stellate cells that play a central role in the pathogenesis of hepatic fibrosis^28^. However, human αSMA expression was detected with a perivascular distribution within the thickened intimal vessel wall in both mildly fibrotic and cirrhotic transplanted animals which is not the normal distribution of hepatic stellate cells. Consequently, we speculated that the MSCs might have transdifferentiated into smooth muscle cells (also expressing αSMA), which frequently occupy this location. This pattern of expression suggested neointimal formation rather than transdifferentiation into activated myofibroblast-like cells. This finding was supported by both the reduction in fibrosis level which was found after MSC transplantation and the absence of any correlation between αSMA expression and fibrosis level. These results were supported by previous studies that demonstrated that bone marrow MSCs are prone to participating in neointimal growth by differentiating into vascular smooth muscle cells expressing the contractile αSMA marker^36^.

In conclusion, MSC transplantation is a beneficial therapeutic strategy in chronic liver diseases on behalf of its anti-inflammmatory, antifibrogenic and transdifferentiation potentials. However, macrophages, as a key determinant component in the stem cell niche, were found to interfere with these functions, necessitating prior manipulation of the microenvironment in which stem cells are engrafted to improve the outcome.
